# Patient satisfaction after breast reconstruction in a high-risk breast cancer population

**DOI:** 10.1016/j.jpra.2025.11.014

**Published:** 2025-11-21

**Authors:** Juliënne A. Berben, Esther M. Heuts, Andrzej A. Piatkowski de Grzymala, Renée M. Miseré, Encarnacion B. Gómez García, Sander M.J. van Kuijk, Hebon, René R.W.J. van der Hulst

**Affiliations:** a Department of Plastic, Reconstructive, and Hand Surgery, Maastricht University Medical Center+, Maastricht, The Netherlands; b NUTRIM School of Nutrition and Translational Research in Metabolism, Maastricht University, Maastricht, The Netherlands; c Department of General Surgery, Maastricht University Medical Center+, Maastricht, The Netherlands; d Department of Clinical Genetics, Maastricht University Medical Center+, Maastricht, The Netherlands; e Netherlands Cancer Institute, The Hereditary Breast and Ovarian Cancer Research Group Netherlands (HEBON), Amsterdam, The Netherlands; f Department of Clinical Epidemiology and Medical Technology Assessment, Maastricht University Medical Center+, Maastricht, The Netherlands

**Keywords:** Breast cancer, Risk reducing mastectomy, Prophylactic mastectomy, Genetic predisposition, *BRCA-1/2* Breast reconstruction, Quality of life

## Abstract

**Background:**

Women with an elevated risk for breast cancer due to a *BRCA*1 or *BRCA*2 pathogenic variant can choose for surveillance or risk reducing mastectomy (RRM). Breast reconstruction improves quality of life (QoL). However, in this high-risk population, the effect of breast reconstruction on QoL nor the level of satisfaction afterward is widely researched. This cross-sectional study aimed to determine the long-term satisfaction level with breast reconstruction in a high-risk breast cancer population.

**Method:**

Women who underwent breast reconstruction were invited to complete several online questionnaires including the BREAST-Q, SF-36, general questions on their health and DNA test results, and the course of diagnosis and treatment. In the analyses two groups were compared: patients with breast reconstruction after risk-reducing mastectomy (BRaRRM) and patients with breast reconstruction after breast cancer (BRaBC).

**Results:**

About 935 participants completed all questionnaires of whom 535 underwent breast reconstruction and were included in this analysis. There was no difference between BREAST-Q “satisfaction with outcome” scales between the BRaRRM and BRaBC groups (60.9 vs 60.9). According to our multivariable linear regression analysis, higher age had a positive association with satisfaction with outcome, and both secondary and implant-based reconstruction were negatively associated with satisfaction with outcome (*b* = 0.21, *b* = −2.10, *b* = −5.83).

**Conclusion:**

RRM combined with breast reconstruction is a good option for women with high risk of breast cancer. No difference in the BREAST-Q satisfaction with outcome scale was observed between both groups. Age, type, and timing of reconstruction should be considered when deciding on RRM.

## Introduction

Breast cancer is the most common cancer in women worldwide and represents 29.4 % of all cancer cases in the female population.^1^ Some women have an elevated risk of developing breast cancer due to an inheritable genetic predisposition. It is estimated that 5–10 % of all breast cancers occur in patients with a hereditary cancer syndrome (HCS). Among all HCSs *BRCA1* and *BRCA2* pathogenic variants are the best known and extensively researched. Pathogenic variations in these two genes result in a 69–72 % risk of developing breast cancer up to age 80.^2^ Other DNA repair genes associated with HCSs and increased risk of breast cancer are *CHEK2, PALB2, ATM*, BARD1, RAD51C, RAD51D, and TP53.^3^ Women with a *moderate* or high risk of developing breast cancer have several options for risk management including screening and risk-reducing mastectomy (RRM). Screening options depend on the country of origin, the gene where the variant is located, and the associated risk level for breast cancer. Screening usually consists of mammography, sometimes combined with magnetic resonance imaging (MRI).^4^^,^^5^ When opting for RRM, women are faced with several choices. RRM can be executed skin or nipple-sparing and optionally followed by breast reconstruction. Options for breast reconstruction can vary both in timing and on the type of reconstruction.^6^^,^^7^

Over the past 25 years, advancements in genetic testing and the implementation of risk-reducing interventions have substantially enhanced the understanding of the prognostic impacts of these measures. Despite the benefits of screening, it does not decrease the risk of developing breast cancer. Early detection through screening, however, is associated with improved survival rates. Nonetheless, patients may still require surgery and possibly additional cancer treatments, which come with their own side effects. RRM can lower the risk of breast cancer by up to 90 %, alongside a significant decrease in associated anxiety and psychological distress.^4^

Nonetheless, RRM does not completely eliminate the risk, as residual breast tissue leaves a small chance of developing cancer.^8^ Additionally, RRM is a surgical procedure that may involve complications and breast reconstruction might not be possible, have side effects, or could fail due to complications. Despite these considerations, it seems that women who choose RRM are willing to accept the potential adverse effects to alleviate the psychological burden of breast cancer risk.^9^

To our knowledge, there are few studies on satisfaction with RRM and subsequent breast reconstruction in women with an elevated risk of breast cancer. Particularly, long-term outcomes in large cohorts are lacking in the literature.^9^

Historically, cancer treatment has predominantly focused on survival; however, with improved survival rates, the emphasis in both treatment and research has increasingly shifted toward long-term outcomes, such as functionality and quality of life (QoL) after cancer.^10^ It remains unclear whether the results of QoL surveys for breast cancer patients also apply to women with a genetic predisposition. The psychological impact of being at elevated risk for breast and possibly other cancers, along with the experience of preventive surgery, could influence QoL outcomes. Therefore, this cross-sectional study aimed to determine the long-term satisfaction level with breast reconstruction in a high-risk breast cancer population and to assess which patient characteristics are associated with this level of satisfaction.

## Methods

### Data collection

For this cross-sectional study, women who had given informed consent to participate in a nationwide study [HEreditary Breast and Ovarian cancer study, the Netherlands (HEBON study)] were invited to participate. Participants of the HEBON study are women who underwent genetic screening for any of the hereditary breast and/or ovarian pathogenic variants.^11^

For this study, women with a proven *BRCA1/2* genetic mutation and women who opted for RRM regardless of the DNA test result were selected from the HEBON database. Those who consented were eligible for participation and were invited to fill in an online questionnaire (Qualtrics, Provo, UT) through a letter. A reminder was sent after three weeks.

For this study, only those women who underwent breast reconstruction were selected. For some analyses, they were divided into two groups: patients with breast reconstruction after risk-reducing mastectomy (BRaRRM) and patients with breast reconstruction after breast cancer (BRaBC). Patients with either ductal carcinoma in situ (DCIS) or invasive mamma carcinoma were also assigned to the BRaBC group in the case of a RRM on the contralateral side.

The online questionnaire included a general questionnaire on patient history and experience, the BREAST-Q for breast-related QoL, BODY-Q for body-related QoL, and the SF-36 for health-related QoL. Furthermore, all participants filled in a physical complaint score, however, these outcomes were not used in this current part of the study.

This study was approved by the Medical Ethics Review Committee of AZM/Maastricht University (METC 2021-2837) and carried out in accordance with the World Medical Association Declaration of Helsinki.

### BREAST-Q and BODY-Q

The BREAST-Q and BODY-Q are both extensively researched and widely used patient-reported outcomes measures (PROMs) for breast and body-related QoL, including patient satisfaction. For breast-related QoL, the following BREAST-Q domains were used: “Psychosocial Well-being,” “Physical Well-being,” and “Sexual Well-being.” Furthermore, the following domains for satisfaction were used: “Satisfaction with Outcome,” “Implant Look,” and “Implant Feel.” The reconstruction module of the BREAST-Q was used for patients with a bilateral mastectomy with bilateral breast reconstruction. In the case of unilateral breast reconstruction, combined with any other surgery on the contralateral side, the mastectomy module was used. Both modules contain an almost identical “Satisfaction with Outcome” domain. The results for the ‘Satisfaction with Outcome’ domain were therefore combined even though the mastectomy module has fewer questions in this domain. The specific scoring modules were used for obtaining a normalized 0–100 score. For body-related QoL, the BODY-Q “Body Image” and “Body Satisfaction” domains were used. Both the BREAST-Q and the BODY-Q result in a score between 0–100, with a higher score indicating a better breast-related QoL. Scoring differences between outcomes are to be interpreted as clinically relevant when ≥ 4.0.^12^ Additionally, these results were compared with normative data from a general Western European female population.^13^

### SF-36

The SF-36 is a validated PROM used for health-related QoL. It consists of 36 questions distributed over four physical domains (General Health, Physical Functioning, Physical Limitation, and Bodily Pain) and four mental domains (Energy, Social Functioning, Emotional Well-being, and Emotional Limitation). The sum of the scores in the physical domains results in the “Physical Component Scale” and the sum of the mental domains results in the “Mental Component Scale.” All results are translated in a score between 0 and 100, with a higher score indicating a better health-related QoL.^14^ Again, these results were compared with normative data from a general Western European female population.^15^

### Results over time

To visualize different results and clinical characteristics over time, four timeframes for breast reconstruction were created (<1995, 1996–2005, 2006–2015, >2016). BREAST-Q Satisfaction with Outcome scores were calculated for each timeframe. Additionally, the type and timing of reconstruction were counted for each period.

### Statistical analyses

Patient demographics and clinical characteristics were analyzed with descriptive statistics. BRaRRM and BRaBC groups were compared using the independent-samples t-test and Pearson’s chi-square test.

Satisfaction with breast reconstruction was described as mean scores with standard deviation (SD).

Univariate linear regression was used to estimate the association between patient characteristics and satisfaction with breast reconstruction. After this, a multivariable linear regression model was used to determine what influential variables persisted after correction for other independent variables. The following variables were added to the model: age at the time of the reconstruction, BMI, time since reconstruction, indication of mastectomy (risk-reducing or therapeutic), autonomy in the decision, timing of reconstruction (primary or secondary), type of reconstruction, complications, and current cup size. Radiotherapy and systemic therapy were not added to the multivariable linear regression model since no patients in the RRM group underwent radiotherapy. Therefore, by adding the indication for the mastectomy as a potential confounder, there was already corrected for the potential effect of radiotherapy or any other breast cancer therapy.

## Results

In total 935 patients completed all questionnaires, of whom 535 had a breast reconstruction and were included in this analysis. A comprehensive report on the inclusion can be found in Appendix A. The BRaRRM and BRaBC groups differed in both age at inclusion (54.4 ± 10.0 vs. 60.8 ± 8.7, *p* < .001) and age at the time of reconstruction (41.7 ± 9.4 vs. 47.6 ± 9.2, *p* < .001). Patients with BRaBC scored 0.8 (*p* < .001) points higher on a Likert scale of 1 to 5, with one meaning full autonomy and five no autonomy, for autonomy during the decision-making process for RRM, indicating that they had less autonomy than women with BRaRRM. There was also a significant difference in timing and type of reconstruction between the two groups, with the BRaRRM group more often undergoing primary and implant-based reconstruction (*p* < .001). All demographic and clinical characteristics are presented in Table 1.

**Table 1:** Demographic and clinical characteristics.

	BRaRRM (*n* = 250)	BRaBC (*n* = 285)	CI	*p*-value
Age at inclusion (mean, SD)	54.4 ±10.0	60.8 ±8.7	-8.02 – -4.84	<.001
Age at the time of reconstruction (mean, SD)	41.7 ±9.4	47.6 ±9.2	-7.52 – -4.31	<.001
BMI (kg/m2)	25.8 ±4.6	25.5 ±4.3	-0.43 – 1.08	.40
Smoking				.12
Yes (n, %)	12 (4.8)	15 (5.3)		
No, quit (n, %)	98 (39.2)	136 (47.7)		
No, never (n, %)	139 (55.6)	134 (47.0)		
Children (n, %)	211 (84.4)	241 (84.6)		.96
Partner (n, %)	216 (86.4)	241 (84.6)		.55
Employment (n, %)				<.001
Yes	190 (76.0)	158 (55.4)		
No, pension	33 (13.2)	79 (27.7)		
No	27 (10.8)	48 (16.8)		
Proven genetic mutation (n, %)	249 (99.6)	217 (76.1)		<.001
BRCA1	164 (65.6)	135 (47.4)		.07
BRCA2	86 (34.4)	75 (26.3)		
CHEK2	0	3 (1.1)		
Other/unknown	0	7 (2.8)		
Educational Attainment				
Primary (n, %)	1 (0.4)	2 (0.7)		.72
Secondary (n, %)	113 (45.2)	143 (50.2)		.14
Bachelors (n, %)	98 (39.2)	117 (41.1)		
Masters (n, %)	38 (15.2)	23 (8.1)		
Allergies (n, %)	87(34.8)	95 (33.3)		
Chronic disease (n, %)	82 (32.8)	111 (38.9)		
Diabetes (n, %)	4 (6.4)	12 (4.2)		
Asthma/COPD (n, %)	16 (16.4)	20 (7.0)		
Coronary disease (n, %)	14 (5.6)	31 (10.9)		
Auto-immune disease (n, %)	17 (6.8)	14 (4.9)		
Fibromyalgia (n, %)	14 (5.6)	8 (2.8)	-0.12 – 1.67	.09
IBS (n, %)	12 (4.8)	8 (2.8)	-1.64 – 0.56	.34
Thyroid disease (n, %)	15 (6.0)	26 (9.1)		
Other (n, %)	27 (10.8)	36 (12.6)		
Time since DNA test (mean years, SD)	18.4 ±4.7	17.6 ±5.7		
Time since Breast Reconstruction (mean years, SD)	12.6 ±5.7	13.1 ±6.9		
Autonomy during decision (mean, SD)	1.3 ±0.6	2.1 ±1.2	-0.97 – -0.64	<.001
Full autonomy (n, %)	197 (78.8)	127 (44.6)		
Full autonomy with consideration of physician’s opinion (n, %)	39 (15.6)	61 (21.4)		
Decision together with physician (n, %)	11 (4.4)	59 (20.7)		
Physician decided with consideration of patient’s opinion (n, %)	2 (0.8)	21 (7.4)		
Physician decided (n, %)	1 (0.4)	17 (6.0)		
Cup size (EU), pre-operative (mean)	C	C		.90
AA (n, %)	6 (2.4)	3 (1.1)		
A (n, %)	31 (12.4)	21 (7.4)		
B (n, %)	78 (31.2)	103 (36.1)		
C (n, %)	57 (22.8)	84 (29.5)		
D (n, %)	44 (17.6)	43 (15.1)		
DD/E (n, %)	24 (9.6)	19 (6.7)		
>E (n, %)	10 (4.0)	12 (4.2)		
Cup size (EU), post-operative (mean)	B-C	B-C		.28
AA (n, %)	10 (4.0)	19 (6.7)		
A (n, %)	14 (5.6)	10 (3.5)		
B (n, %)	97 (38.8)	120 (42.1)		
C (n, %)	85 (34.0)	95 (33.3)		
D (n, %)	33 (13.2)	30 (10.5)		
DD/E (n, %)	10 (4.0)	8 (2.8)		
>E (n, %)	1 (0.4)	3 (1.1)		
Implant-based Reconstruction (n, %)	172 (68.8)	145 (50.9)		<.001
Primary breast reconstruction (n, %)	195 (78.0)	112 (39.3)		<.001
Secondary breast reconstruction (n, %)	55 (22.0)	124 (43.5)		<.001
Combination of the two (n, %)	-	49 (17.2)		<.001
Radiotherapy (n, %)	-	130 (45.6)		<.001
Complications	76 (30.4)	109 (38.2)		.06
Infection (n, %)	35 (14.0)	58 (20.4)		
Hematoma/bleeding (n, %)	12 (4.8)	18 (6.3)		
Seroma (n, %)	10 (4.0)	16 (5.6)		
Wound healing (n, %)	19 (7.6)	31 (10.9)		
Necrosis (n, %)	15 (6.0)	34 (11.9)		
Flap loss (n, %)	3 (1.2)	6 (2.1)		
Implant loss (n, %)	30 (12.0)	37 (13.0		
Other (n, %)	18 (7.2)	20 (7.0)		

**Figure 1:**
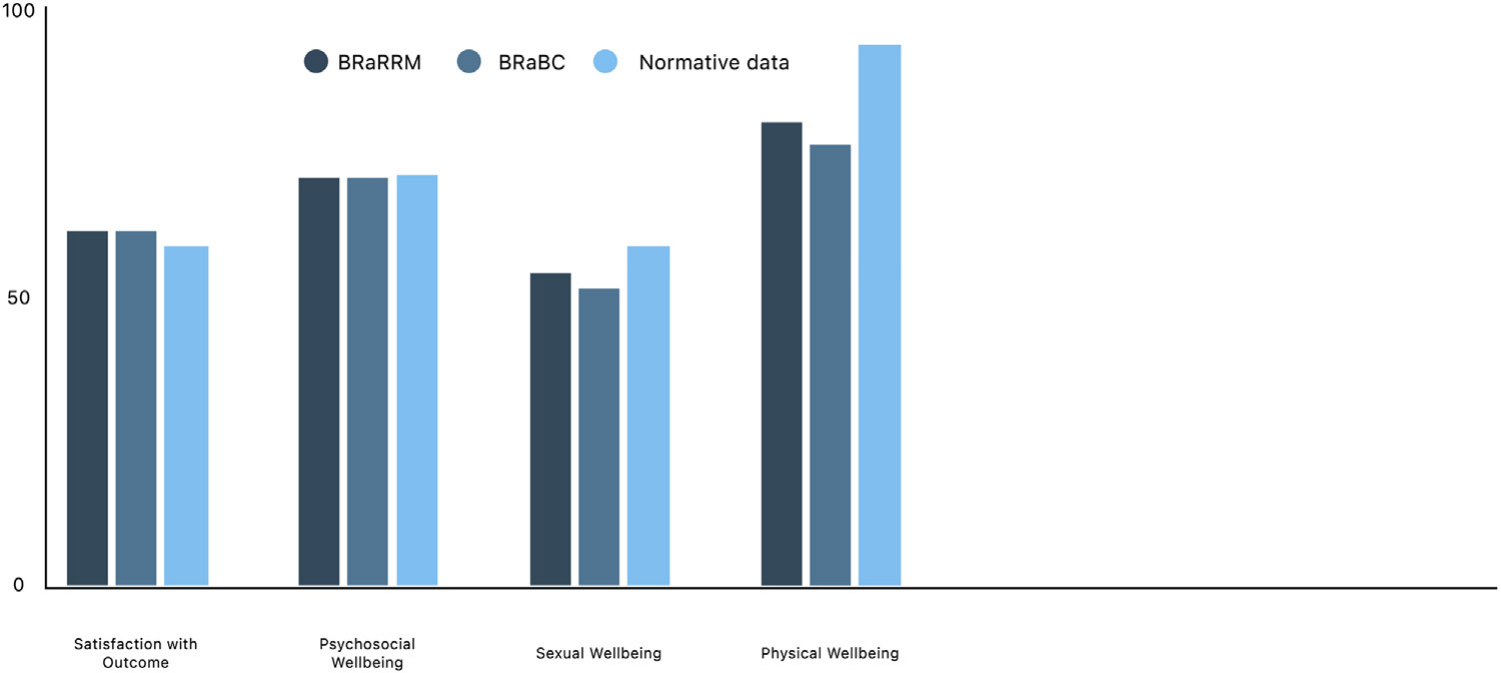
BREAST-Q outcomes for BRaRRM and BRaBC including normative data.

**Figure 2:**
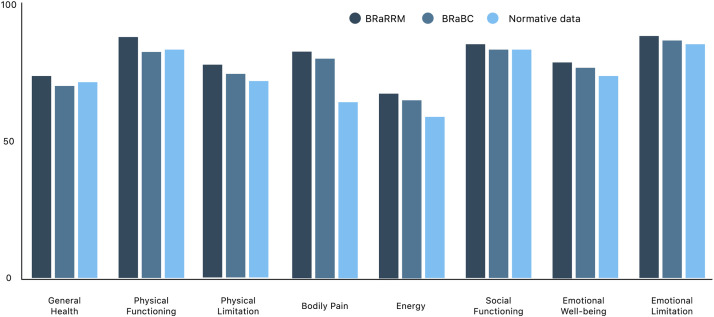
SF-36 outcomes for BRaRRM and BRaBC including normative data.

The mean outcome of the BREAST-Q “Satisfaction with outcome” scale for all participants was 60.9. Additionally, there was no difference in satisfaction with breast reconstruction when comparing the two groups (60.9 vs 60.9). Only the BREAST-Q scales “Sexual wellbeing” (54.3 vs. 51.1, *p* = .04) and “Physical wellbeing” (79.6 vs. 75.8, *p* = .03) had a difference with the BRaRRM group scoring higher. Both groups show BREAST-Q outcomes similar to normative data except for the “Physical well-being” scale and “Sexual well-being” scale, as shown in Figure 1.^13^ The BRaRRM group also scored higher than the BRaBC group on some of the physical scales of the SF-36, including the Physical Component Scale (80.5 vs. 76.1, *p* = .01). All mean differences in PROM outcomes are presented in Table 2. Figure 2 shows the SF-36 scales for both groups compared to normative data, visualizing the minor between-group differences.^15^

**Table 2:** Mean differences in PROM outcomes.

	BRaRRM (*n* = 250)	BRaBC (*n* = 285)	Mean difference	CI	*p*-value
SF-3H6					
General health	73.0 ± 18.9	69.4 ± 20.7	3.6	0.18 to 6.99	.04
Physical functioning	87.1 ± 16.0	81.8 ± 19.6	4.4	1.03 to 7.69	.01
Physical limitation	79.6 ± 35.1	73.6 ± 38.0	6.0	−0.23 to 12.3	.06
Bodily pain	81.9 ± 18.8	79.6 ± 20.7	2.3	−1.07 to 5.70	.18
Physical Component Scale	80.5 ± 18.5	76.1 ± 20.4	4.4	1.03 to 7.69	.01
Energy	66.3 ± 16.9	64.5 ± 18.2	1.7	−1.26 to 4.75	.26
Social functioning	84.6 ± 21.3	83.5 ± 20.4	1.0	−2.52 to 4.57	.57
Emotional well-being	78.2 ± 13.8	76.2 ± 15.1	2.0	−0.51 to 4.45	.12
Emotional limitation	87.6 ± 28.8	86.0 ± 32.4	1.6	−3.65 to 6.85	.55
Mental Component Scale	79.1 ± 17.0	77.6 ± 17.8	1.5	−1.45 to 4.48	.32
BREAST-Q					
Satisfaction with outcome	60.9 ± 17.3	60.9 ± 18.8	0.0	−3.09 to 3.18	.98
Sexual wellbeing	54.3 ± 17.2	51.1 ± 17.6	3.2	0.23 to 6.16	.04
Psychosocial	70.2 ± 17.1	70.0 ± 18.5	0.2	−2.84 to 3.23	.90
Physical wellbeing	79.7 ± 20.1	75.8 ± 21.0	3.9	0.35 to 7.36	.03
Implant look	(*n* = 175)	(*n* = 161	1.7	−7.52 to 4.16	.57
Implant feel	65.7 ± 27.3	67.4 ± 27.1	0.5	−6.08 to 5.03	.85
Satisfaction with outcome	66.7 ± 26.3	67.2 ± 25.3			
BODY-Q					
Body image	60.2 ± 23.6	59.4 ± 22.4	0.8	−3.12 to 4.69	.69
Body satisfaction	57.0 ± 21.5	58.4 ± 20.1	1.4	−4.93 to 2.14	.44

Table 3 presents the regression coefficients for potential influencers of the BREAST-Q “Satisfaction with outcome” scale. Most characteristics tested with univariate regression persisted after correction with multivariable regression except for time since breast reconstruction (*b* = −0.02, 95 % CI: −0.29 to 0.24, *p* = .86). Additionally, BMI does present itself with a negative association in the multivariable regression analysis (−0.59, 95 % CI: −0.79 to −0.09, *p* = <.05). The following variables were associated with satisfaction with outcome: Age at the time of reconstruction (*b* = 0.21 per year, 95 % CI: 0.03–0.38, *p* ≤ .05), Timing of Reconstruction with secondary reconstruction scoring lower on satisfaction (*b* = −2.79, 95 % CI: −5.33 to −0.25, *p* = <.05), complications (*b* = −5.50, 95 % CI: −8.73 to −2.26, *p* = <.001), Type of Reconstruction with implant-based scoring lower on satisfaction (*b* = −6.65, 95 % CI: −9.80 to −3.51, *p* = <.001), and Current Cup size (*b* = 3.47 per size, 95 % CI: 1.95–4.99, *p* = <.001) with a larger cup size resulting in higher satisfaction. There is a mean difference in autonomy during decision-making between BRaRRM and BRaBC. However, when looking at linear regression outcomes, less autonomy during the decision-making process only seems to have a small, not statistically significant, negative effect on satisfaction with outcome (*b* = −1.00, *p* =.18 and *b* = −1.34, *p* =.09).

**Table 3:** Regression coefficients for BREAST-Q satisfaction with outcome.

BREAST-Q	Satisfaction with outcome					
	Linear regression (b, CI, *p*-value)			Multivariable linear regression (b, CI, *p*-value)		
Age at inclusion	0.04	−0.12 to 0.20	.65	-	-	-
Age at the time of Reconstruction	0.17	0.01 to 0.33	<.05	0.21	0.03 to 0.38	<.05
BMI	−0.30	−0.65 to 0.05	.09	−0.59	−0.79 to −0.09	<.05
Time since DNA test	−0.25	−0.55 to 0.06	.12	-	-	-
Time since Breast Reconstruction	−0.29	−0.53 to −0.04	<.05	−0.02	−0.29 to 0.24	.86
Radiotherapy	0.71	−3.05 to 4.47	.71	-	-	-
Proven genetic mutation	−0.75	−5.49 to 4.00	.76	-	-	-
Indication mastectomy	−0.04	−3.18 to 3.09	.98	0.68	−2.95 to 4.30	.72
Autonomy during decision	−1.00	−2.47 to 0.47	.18	−1.34	−2.89 to 0.22	.09
Timing Reconstruction	−2.10	−4.49 to 0.28	.08	−2.79	−5.33 to −0.25	<.05
Complication(s)	−5.67	−8.96 to −2.38	<.001	−5.50	−8.73 to −2.26	<.001
Type of Reconstruction	−5.83	−9.01 to −2.66	<.001	−6.65	−9.80 to −3.51	<.001
Current cup size	2.89	1.42 to 4.36	<.001	3.47	1.95 to 4.99	<.001

**Figure 3:**
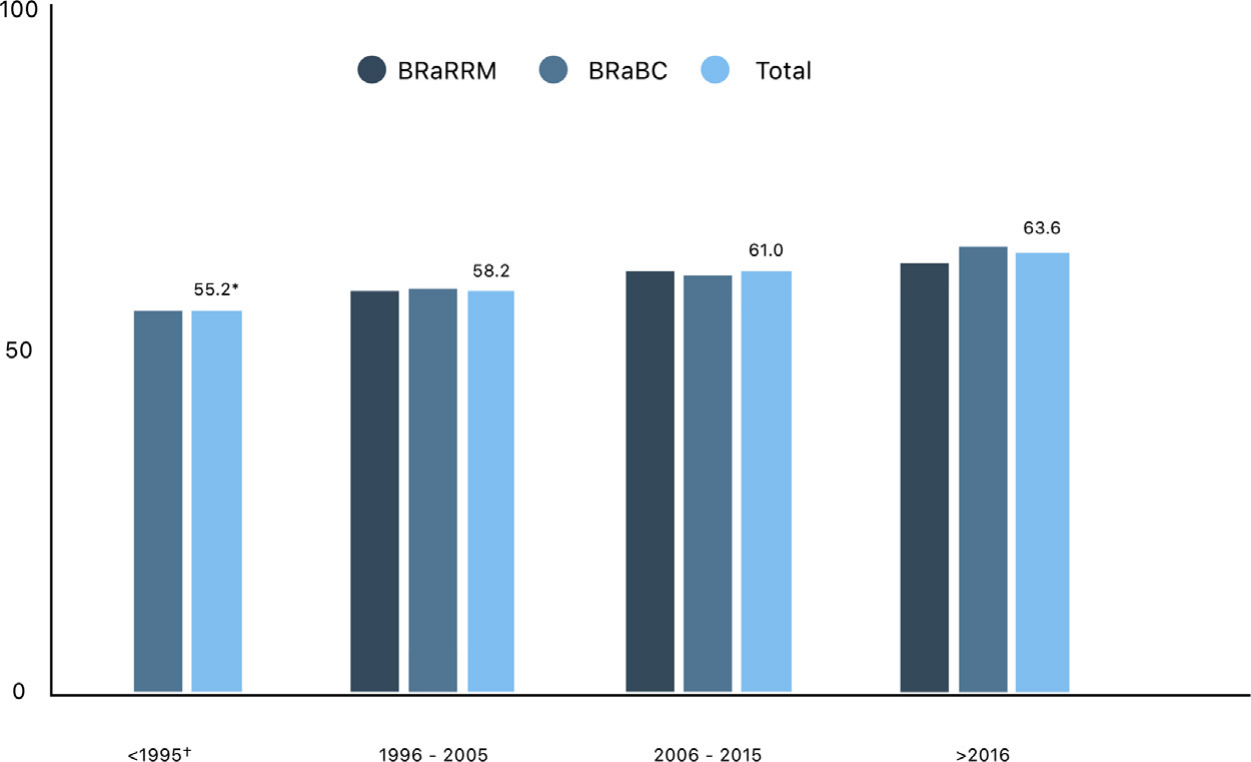
BREAST-Q satisfaction with outcome results over time. †Years indicate the year breast reconstruction took place. * BREAST-Q satisfaction with outcome score for all subjects.

**Figure 4:**
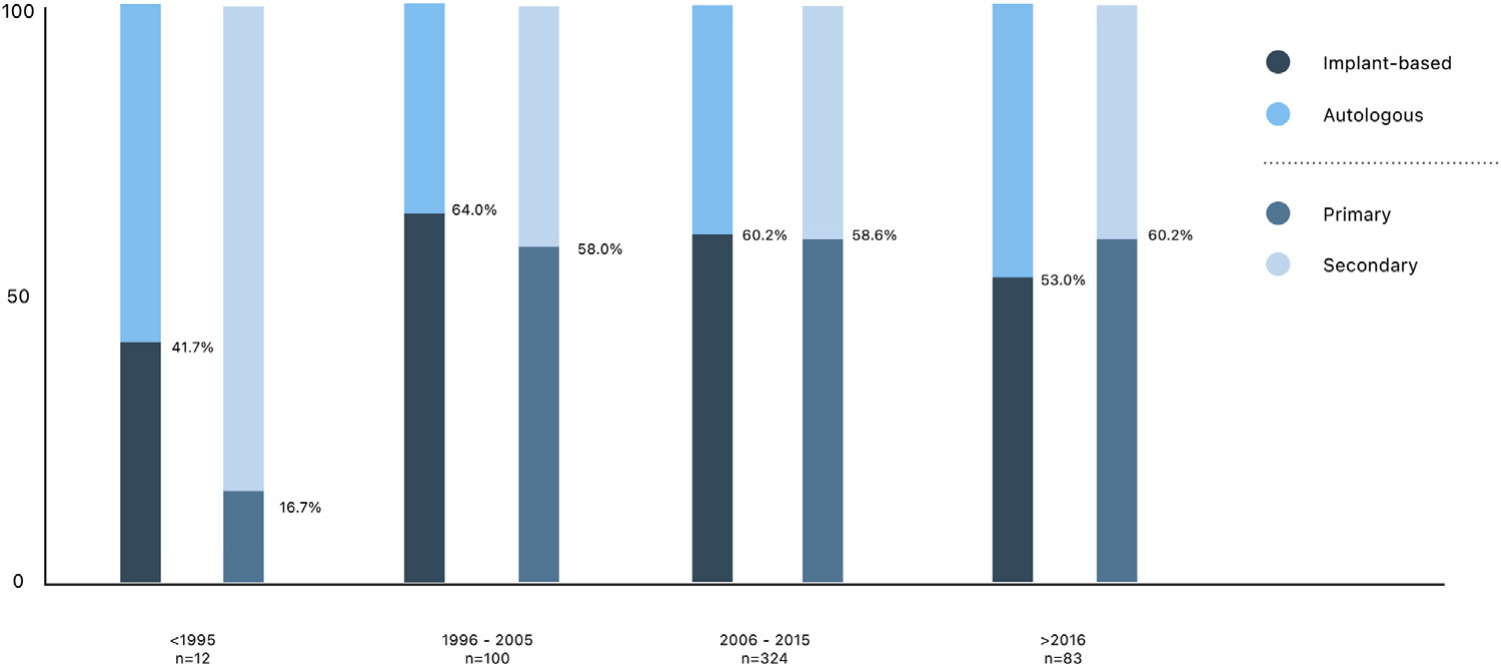
Type of reconstruction and timing of reconstruction over time since 1978. * The bar chart bars present the ratio between implant-based and autologous reconstruction on the left, with the percentage indicating the share of implant-based reconstructions. The bars on every right side present the ratio between primary and secondary reconstruction, with the percentage indicating the share of primary reconstructions. † Dates indicate the year breast reconstruction took place.

The BREAST-Q “Satisfaction with outcome” scale appears to improve among patients who had their reconstruction in a more recent period. As shown in Figure 3, women who had their breast reconstruction before 1995 scored 8.4 points lower on the satisfaction with outcome scale than women who were operated on after 2016 (55.2 vs. 63.6). This figure was merely added to visualize the change over time however, these results were not statistically compared. To visualize a potential cause for this increase in satisfaction Figure 4 was added, presenting the change in type and timing of breast reconstruction over time. It shows an increase in autologous reconstructions as well as primary breast reconstruction. According to the multivariable regression analysis, both autologous and primary reconstruction have a positive effect on satisfaction with outcome.

## Discussion

Since the introduction of genetic testing in high-risk breast cancer patients, the effect of RRM and screening on survival outcomes has been widely researched. With rising long-term survival rates, QoL has become an important factor in this population. However, to our knowledge, no large long-term cohort studies have been conducted on the subject in relation to breast reconstruction. This cross-sectional study aimed to determine the satisfaction level after breast reconstruction in women with a high risk of breast cancer. This resulted in a large dataset with over 500 women who underwent breast reconstruction either after RRM or breast cancer treatment.

Our findings suggest no significant difference in satisfaction levels between women who had breast reconstruction after RRM and those who underwent reconstruction following a mastectomy due to breast cancer. Their satisfaction levels were consistent with normative data, as illustrated in Figure 1 (60.9 vs. 57).^13^ This indicates that living with a high risk of breast cancer and opting for preventive surgery on healthy breast tissue do not negatively impact satisfaction outcomes. Normative data are never collected with the purpose of being compared with one specific study; thus, the heterogeneity between the datasets should be taken into account. However, these results do imply that the satisfaction levels within our population are comparable with a healthy female population.

Our data do, however, show a difference in sexual well-being between normative data and both study groups (58.0 vs 51.1 and 54.3, respectively) (Figure 4). This implies that mastectomy, even with reconstruction, affects sexual well-being, which is to be expected. However, the minor difference between the BRaBC and BRaRRM group is, according to BEAST-Q guidelines, not clinically relevant; it is a difference that can be explained by, for instance, effects of adjuvant therapy. Breast cancer therapy, such as chemotherapy or anti-endocrine therapy, can lead to early menopausal symptoms.^16^ Effects on sexual well-being for both groups should be taken into account when counseling for RRM. It is known that, although counseling for sexual well-being is recommended, health care professionals feel a barrier to implementing sexual well-being in oncological counseling.^17^

The median age for a primary breast cancer diagnosis in the general population is 63 years.^18^ Women with a genetic predisposition for breast cancer are generally diagnosed at a younger age.^19^ This was reflected in our data, where women were notably younger at the time of their breast reconstruction and, in the case of the BRaBC group, their breast cancer diagnosis (41.7 and 47.6 years, respectively).

The multivariable linear regression model revealed that younger age was associated with slightly lower satisfaction levels, even after adjustment. Based on age, one might expect the high-risk population to report lower satisfaction than the normative data, and for the BRaRRM group to score lower than the BRaBC group. However, this was not observed. The model also indicated that autologous reconstruction had a strong positive influence on satisfaction with outcome, consistent with previous studies.^20^ In our data, autologous reconstruction was less common in the BRaRRM group compared to the BRaBC group (31.2 % vs. 49.1 %). This difference might suggest lower satisfaction for the BRaRRM group. However, Santosa et al. found that younger patients with implant-based reconstructions reported higher satisfaction than older ones, whereas age did not affect satisfaction in patients with autologous reconstructions.^21^ This could explain why the BRaRRM group, with its younger age and higher proportion of implant-based reconstructions, reported similar satisfaction levels to the BRaBC group. These findings suggest that when considering RRM with breast reconstruction, age should be taken into consideration when deciding on the type of reconstruction.

It is well-established that the provided information and the degree of autonomy are important during the decision-making process for RRM.^22^^,^^23^ Additionally, genetic counseling before and after testing is essential to aid patients in understanding the potential outcomes and their implications.^24^ Our results showed a difference in perceived autonomy during the decision for RRM between the BRaRRM and BRaBC groups. Women with a breast cancer diagnosis experienced less autonomy during this process compared to those considering bilateral RRM. However, after adjusting for potential confounders, such as the presence of a breast cancer diagnosis, autonomy did not significantly influence satisfaction with breast reconstruction. When faced with a breast cancer diagnosis, patients may prioritize treatment and its timely initiation over personal autonomy, which could explain why autonomy did not impact their satisfaction with the outcome.

Over time, both the type and timing of breast reconstruction as well as the approach to risk reduction in this high-risk population have changed, as shown in Figure 4. Advances in technique and approach seem to have positively influenced satisfaction levels, as is visualized in Figure 3. Our data showed that primary reconstruction also positively affected satisfaction with outcomes in both groups, which contrasts with previous literature.^10^^,^^25^ The current theory is that women who underwent a mastectomy are more satisfied with their breast reconstruction since they have experienced their bodies with a “flat chest.” However, the lower mean age in our population may have contributed to better aesthetic outcomes with primary breast reconstruction. Additionally, advancements in surgical techniques for skin- and nipple-sparing mastectomies have improved aesthetic results when combined with primary breast reconstruction.^26^ Another explanation could be that the fear of getting breast cancer, that was probably part of the motivation for the women in the BRaRRM group, contributes to a higher satisfaction level. The women in this study mainly have a genetic predisposition that leads to a high lifetime risk for breast cancer. International guidelines for other genetic predispositions, with a moderate lifetime risk for breast cancer, differ on whether to offer RRM.^5^ It could be suggested that these groups experience less fear, and ultimately, the level of satisfaction after reconstruction could be affected by this.

The type of autologous flap used for reconstruction and the technique used for implant-based reconstruction have changed over time and have led to different results in breast reconstruction. The same applies to the type of implant used for reconstruction. One of the limitations of our studies was that, due to its retrospective design, very little information on reconstructive technique was available. Adding this information could result in more specific reconstruction advice for this group of patients.

Although the long-term follow-up strengthens our data, it also allows for recall bias. Some women who participated in this study had their genetic screening or breast cancer diagnosis more than two decades ago and may not accurately remember the process of their diagnosis and treatment. This bias applies to both the BRaBC and BRaRRM groups. The length of the participation of a large part of the women in this study implies that these participants are highly motivated. It cannot be excluded that women dropping out of the study did so due to, for instance, complications or lower satisfaction levels. In addition, the BRaBC group is at risk for survivor bias, as long-term cancer survivors, despite previous treatment and possible lingering symptoms, often report higher QoL outcomes.^27^ This might have affected the outcomes of the BRaBC group, resulting in higher outcomes. However, to what extent is unknown.

On the other hand, in elective procedures, such as breast reconstruction, patient expectations play a critical role in their ultimate satisfaction with the result.^28^ Due to this study’s cross-sectional design, no data were available on the baseline QoL of the study participants. Nor did we have any information on their pre-operative expectations. Nevertheless, we believe these results provide a representative view of the current satisfaction levels with breast reconstruction for both groups.

## Conclusion

For women at elevated risk for breast cancer due to a *BRCA1* or *BRCA2* pathogenic variant, RRM can significantly reduce risk while offering a prognosis and life expectancy comparable to that of surveillance, along with reduced fear. When combined with breast reconstruction, QoL levels remain similar to normative data. Our findings indicate that satisfaction with breast reconstruction outcomes is similar for women undergoing the procedure following RRM or breast cancer treatment within this high-risk population. Therefore, RRM combined with breast reconstruction is a suitable option for women with a genetic predisposition to breast cancer. Although the level of autonomy during decision-making appears to be non-influential, it remains an important consideration. Additionally, primary autologous breast reconstruction tends to yield higher satisfaction levels than secondary reconstruction.

## Funding

The HEBON study is supported by the Dutch Cancer Society grants NKI1998-1854, NKI2004-3088, NKI2007-3756, NKI 12535, the Netherlands Organisation of Scientific Research grant NWO 91109024, the Pink Ribbon grants 110005 and 2014-187.WO76, the BBMRI grant NWO 184.021.007/CP46, and the Transcan grant JTC 2012 Cancer 12-054.

## Ethical approval

This study was approved by the Medical Ethics Review Committee of AZM/Maastricht University (METC 2021-2837) and carried out in accordance with the World Medical Association Declaration of Helsinki.

## Declaration of competing interest

The authors declared no potential conflicts of interest with respect to the research, authorship, and publication of this article.

## Appendix Supplementary materials

Image, application 1

## References

[1] European Comission, Breast Cancer in the EU 2022. 2024. Accessed January 11, 2024. https://ecis.jrc.ec.europa.eu/pdf/Breast_cancer_2022-Oct_2023.pdf.

[2] Kuchenbaecker K.B., Hopper J.L., Barnes D.R. (2017). Risks of breast, ovarian, and contralateral breast cancer for BRCA1 and BRCA2 mutation carriers. JAMA.

[3] Dorling L., Carvalho S., Allen J. (2021). Breast cancer risk genes: association analysis in more than 113,000 women. N Engl J Med.

[4] Paluch-Shimon S., Cardoso F., Sessa C. (2016). Prevention and screening in BRCA mutation carriers and other breast/ovarian hereditary cancer syndromes: ESMO Clinical Practice Guidelines for cancer prevention and screening. Ann Oncol.

[5] Nielsen S.M., Eccles D.M., Romero I.L. (2018). Genetic testing and clinical management practices for variants in non-BRCA1/2 breast (and breast/ovarian) cancer susceptibility genes: an international survey by the Evidence-Based Network for the Interpretation of Germline Mutant Alleles (ENIGMA) clinical working group. JCO Precis Oncol.

[6] De Lorenzi F., Borelli F., Catapano S., Alessandri-Bonetti M., Sala P., Veronesi P. (2023). Postmastectomy breast reconstruction for women with hereditary gastric and breast cancer syndrome. Eur J Cancer Prevent.

[7] Aslam A., Arshad Z., Ahmed A. (2023). Bilateral risk-reducing mastectomy and reconstruction: a 12-year review of methodological trends and outcomes at a tertiary referral centre. PLoS One.

[8] Carbine N.E., Lostumbo L., Wallace J., Ko H. (2018). Risk-reducing mastectomy for the prevention of primary breast cancer. Cochr Datab System Rev.

[9] Bresser P.J., Seynaeve C., Van Gool A.R. (2006). Satisfaction with prophylactic mastectomy and breast reconstruction in genetically predisposed women. Plast Reconstr Surg.

[10] Miseré R.M., van Kuijk S.M., Claassens E.L., Heuts E.M., Piatkowski A.A., van der Hulst R.R. (2021). Breast-related and body-related quality of life following autologous breast reconstruction is superior to implant-based breast reconstruction: a long-term follow-up study. Breast.

[11] Pijpe A., Manders P., Brohet R.M. (2010). Physical activity and the risk of breast cancer in BRCA1/2 mutation carriers. Breast Cancer Res Treat.

[12] Cohen W.A., Mundy L.R., Ballard T.N. (2016). The BREAST-Q in surgical research: a review of the literature 2009-2015. J Plast Reconstr Aesthet Surg.

[13] Jepsen C., Paganini A., Hansson E. (2023). Normative BREAST-Q reconstruction scores for satisfaction and well-being of the breasts and potential donor sites: what are Swedish women of the general population satisfied/dissatisfied with?. J Plast Surg Hand Surg.

[14] Ware J., Kosinski M., Keller S. (1994). SF-36 physical and mental health summary scales. User’s Man.

[15] Garratt A.M., Stavem K. (2017). Measurement properties and normative data for the Norwegian SF-36: results from a general population survey. Health Qual Life Outcomes.

[16] Marsh S., Borges V.F., Coons H.L., Afghahi A. (2020). Sexual health after a breast cancer diagnosis in young women: clinical implications for patients and providers. Breast Cancer Res Treat.

[17] Reese J.B., Beach M.C., Smith K.C. (2017). Effective patient-provider communication about sexual concerns in breast cancer: a qualitative study. Support Care Cancer.

[18] National Cancer Institute (2024). Cancer Stat Facts: female breast cancer. https://seer.cancer.gov/statfacts/html/breast.html.

[19] Daly M.B., Rosenthal E., Cummings S. (2023). The association between age at breast cancer diagnosis and prevalence of pathogenic variants. Breast Cancer Res Treat.

[20] Sadok N., Krabbe-Timmerman I.S., Buisman N.H., van Aalst V.C., de Bock G.H., Werker P.M.N. (2023). Short-term quality of life after autologous compared with alloplastic breast reconstruction: a prospective study. Plast Reconstr Surg.

[21] Santosa K.B., Qi J., Kim H.M., Hamill J.B., Pusic A.L., Wilkins E.G. (2016). Effect of patient age on outcomes in breast reconstruction: results from a multicenter prospective study. J Am Coll Surg.

[22] Borgen P.I., Hill A.D., Tran K.N. (1998). Patient regrets after bilateral prophylactic mastectomy. Ann Surg Oncol.

[23] Payne D.K., Biggs C., Tran K.N., Borgen P.I., Massie M.J. (2000). Women's regrets after bilateral prophylactic mastectomy. Ann Surg Oncol.

[24] Petrova D., Cruz M., Sánchez M-J (2022). BRCA1/2 testing for genetic susceptibility to cancer after 25 years: a scoping review and a primer on ethical implications. Breast.

[25] Eltahir Y., Bosma E., Teixeira N., Werker P.M., de Bock G.H. (2020). Satisfaction with cosmetic outcomes of breast reconstruction: investigations into the correlation between the patients’ Breast-Q outcome and the judgment of panels. JPRAS open.

[26] Cheung C. (2024). Recent trends in nipple sparing mastectomy—a narrative review. Ann Breast Surg.

[27] Doege D., Thong M.S.Y., Weißer L. (2021). Health-related quality of life in very long-term cancer survivors 14-24 years post-diagnosis compared to population controls: a population-based study. Cancers (Basel).

[28] Pusic A.L., Klassen A.F., Snell L. (2012). Measuring and managing patient expectations for breast reconstruction: impact on quality of life and patient satisfaction. Expert Rev Pharmacoecon Outcomes Res.

