# Supplementary figures and images for: Patient satisfaction after breast reconstruction in a high-risk breast cancer population
Source: JPRAS Open. 2025 Nov 21;48:193–204. doi: 10.1016/j.jpra.2025.11.014 (PMC12721067; doi:10.1016/j.jpra.2025.11.014)

**APPENDIX A Flowchart Patient Inclusion**

**
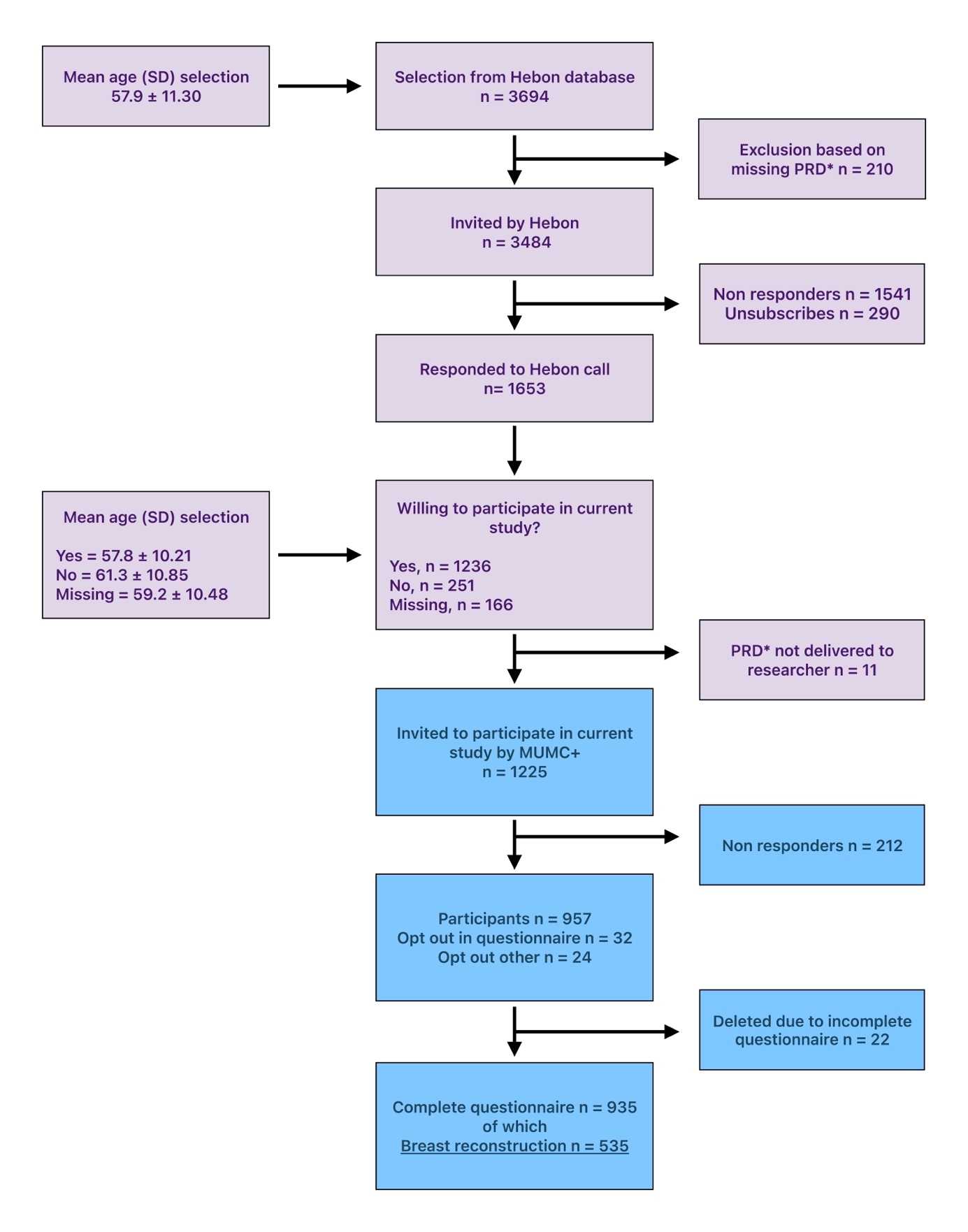
**

* PRD = Personal Records Database

Supplement: Supplementary file 1 [file mmc1.docx]
